# Characterization of *Leishmania* Species Isolated from Cutaneous Human Samples from Central Region of Syria by RFLP Analysis

**DOI:** 10.5402/2013/308726

**Published:** 2013-09-19

**Authors:** Samar Anis Al-Nahhas, Rania Magdy Kaldas

**Affiliations:** ^1^Department of Animal Biology, Faculty of Science, Damascus University, Damascus 96311, Syria; ^2^Vector Biology Research Program, United States Naval Medical Research Unit No. 3 (NAMRU-3), Cairo 11517, Egypt

## Abstract

Cutaneous leishmaniasis (CL) is an endemic disease and a public health problem in Hama governorate located in the central region of Syria. The aim of this study was to characterize *Leishmania* species isolated from human skin samples. A polymerase chain reaction, restriction fragment length polymorphism (PCR-RFLP) assay, was performed on skin lesion material samples from 32 patients with confirmed CL by direct microscopic examination in order to prove its usefulness and efficiency for identification of *Leishmania* species. *Leishmania tropica* (*L. tropica*) is confirmed as an etiologic agent of CL in this area.

## 1. Introduction 

Leishmaniasis is a parasitic endemic disease that spreads over the world in tropical and subtropical regions. Cutaneous leishmaniasis (CL) is currently endemic in 87 countries worldwide (20 countries in the new world and 67 countries in the old world), with an estimation of new infected cases from 500,000 to one million annually [1]. 

In Syria, CL has two forms: zoonotic cutaneous leishmaniasis (ZCL) caused by *L. major* and anthroponotic cutaneous leishmaniasis (ACL) caused by *L. tropica* [2–4]. During the last decade, CL represented a major public health problem in Syria, because the incidence of CL has increased significantly. According to the Syrian Ministry of Health, Department of Infectious Diseases, the reported number of cutaneous leishmaniasis was 12,832 cases in 1999, and this number reached 46,148 cases in 2009. An average of 24% of the reported cases in Syria originated from the middle region (Hama, Idleb, and Homs governorates).

 Traditionally, diagnosis of leishmaniasis in Syria relies on the clinical manifestations of the disease with the detection of the intracellular stages of the parasite by direct examination of smears or biopsies of skin lesions and culturing of specimens [5, 6]. 

However, the *Leishmania* species identification is not possible using these methods because all *Leishmania* parasites are morphologically similar. Actually, isoenzyme analysis is the golden method for identification of *Leishmania* species and subspecies. However, this procedure is time consuming, culture dependent and requires considerable expertise [7–11].

Therefore, accurate diagnosis of cutaneous leishmaniasis, treatment, disease prevention, and controlling strategies, as well as management decisions, require identification of the causative species of *Leishmania* parasite [10–12]. In the last decade, several PCR assays for detection and differentiation of parasites, including species-specific PCR, single-strand conformation polymorphisms (SSCP), and restriction fragment length polymorphism (RFLP) analysis, proved to be sensitive and powerful tools for direct detection of *Leishmania* in clinical samples as well as for parasite characterization [12–14]. A universal PCR method targeting the internal transcribed spacer-1 (ITS1) region lying between the genes coding for 18S rRNA and 5.8S rRNA proved to be useful for direct diagnosis and identification of *Leishmania* parasite, due to its high conservation among species [11, 15–19]. ITS1-PCR and RFLP can be used for direct species identification of *Leishmania* in patient tissues, blood, or other samples preventing the use of microscopic examination and cultivation [15, 16, 18–20]. 

In this study, we aimed to identify the *Leishmania* species that caused cutaneous leishmaniasis in Hama governorate, middle region of Syria.

## 2. Materials and Methods

### 2.1. Samples

**Figure 1:**
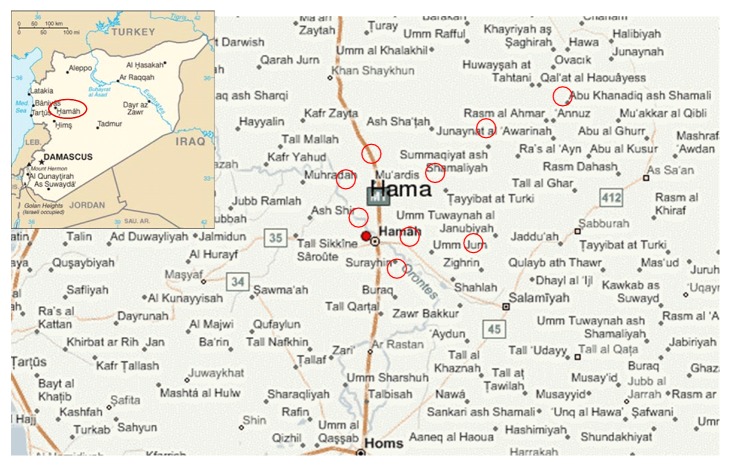
Map showing the areas (circle) in Hama governorate where cutaneous leishmaniasis samples were collected.

A total of 32 samples were collected (between December 2009 and January 2010) from individuals presented to the local sanitary (*Leishmania*) centers for consultation and treatment in Hama, suffering from skin lesions for more than 3 weeks (Figure 1).

 Simple direct questionnaire about clinical and epidemiological information (including age, gender, clinical symptoms, animals, and insects presence) was filled out for each patient. The patients were notified of all the procedures to take place, and a signed informed consent was given to each. The ethics committee of the Ministry of Health, Damascus, Syria, approved this study. After cleaning the lesion, aspirate of skin lesion was smeared, fixed (using methanol), stained (using Giemsa), and examined microscopically for the presence of *Leishmania* amastigotes.

### 2.2. DNA Extraction

Total DNA was extracted from human lesion smear samples using the Qiagen DNA Mini Kit (Qiagen, Valencia, CA), as per tissue protocol instructions, and eluted in 200 *μ*L elution buffer. DNA extracts were stored at −20°C until being used.

### 2.3. PCR Amplification

The internal transcribed region (ITS1) of the small subunit ribosomal DNA was amplified from samples by conventional PCR using the primers L5.8S: 5′-TGATACCACTTATCGCACTT-3′ and LITSR: 5′-CTGGATCATTTTCCGATG-3′; [21].

Amplification reactions were performed in volumes of 50 *μ*L. All PCR assays were optimized with regard to annealing temperature, to concentrations of primers, and to cycling protocols. For all experiments, five *μ*L of isolated DNA was added to the PCR mixture (the mixture contained Go Taq Flexi DNA Polymerase “Promega, USA”: 10 *μ*L Green Go Taq Flexi DNA Polymerase, 3 U Go Taq DNA polymerase, 200 *μ*L dNTPs, 4.0 mM MgCl_2_, and 30 *μ*m each primer “Sigma: Genosys Corp., USA”), and each individual PCR experiment included at least one positive control: *L. tropica* MHOM/TR/05/EP119; *L. major* IPAP/EG89/SI-177 and *L. infantum* EP 50 (5 *μ*L of DNA of reference strain) and one negative control (5 *μ*L of nuclease-free water). The cycling conditions were (94°, 30 sec; 53°, 1 min; 72°, 1 min) repeated for 37 cycles. Amplification products were subjected to electrophoresis in 2% agarose (Sigma-Aldrich, St. Louis, MO) at 100 V in 1x TAE (40 mM Tris-acetate, 1 mM EDTA, pH 8.3) buffer, stained with ethidium bromide (5 *μ*L/100 mL), and visualized and photographed using a UV transilluminator. 

### 2.4. RFLP Analysis of Amplified ITS1

Restriction fragment length polymorphism (RFLP) analysis of the ITS1 amplicons was performed on the ITS1 amplicons, obtained from 32 smear samples and the reference strain, using the restriction enzyme HaeIII (1 *μ*L) (Promega, USA) without prior purification. The digested fragments were subjected to electrophoresis in 2% agarose (Sigma-Aldrich, St. Louis, MO) at 100 V in 1x TAE buffer, stained with ethidium bromide (5 *μ*L/100 mL), and visualized and photographed using a UV transilluminator.

## 3. Results

### 3.1. Characteristics of Patients with Suspected CL

Of the 32 CL cases, 18 (56.3%) were males and 14 (43.8%) were females. The patients were in the age range from 2 to 70 years. None of the examined patients had been out of Hama governorate during the 6 months preceding the onset of lesions, suggesting that these cases are autochthonous. The lesions were mainly located on the upper extremities (67.5%) compared with 25.9% on the facial region and 6.5% on the legs, typical exposed fly bites areas. Most of these patients had more than one lesion (average 1–4 lesions). All patients had localized and typical lesion of CL, from crusted nodule to ulcerated lesions. The lesions diameter varied from 0.2 to 3 cm, and the duration lesion appearance varied from 15 days to 4 months.

### 3.2. *Leishmania* Species Identity

**Figure 2:**
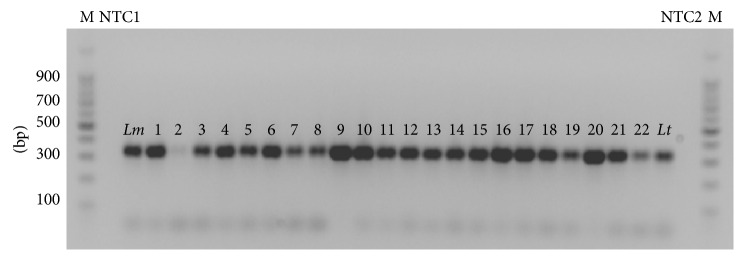
Agarose gel electrophoresis results of ITS1-PCR from Giemsa-stained human smears. M: molecular marker (100 bp); Lanes 1–22: samples; *Lm*: *L. major* (IPAP/EG89/SI-177); *Lt*: *L. tropica* (MHOM/TR/05/EP119) positive control; NTC1, NTC2: negative control for contamination detection.

32 samples (100%) were positive for the presence of *Leishmania*, by ITS1-PCR. The undigested ITS1 amplicons produced a band of expected size depending on whether *Leishmania* species (300–350 bp), when examined by 2% Agarose gel electrophoresis (Figure 2).

**Figure 3:**
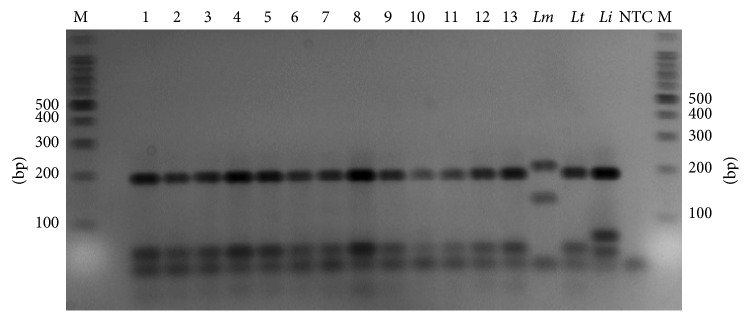
Restriction fragment length polymorphism (RFLP) analysis of ITS1-PCR fragments amplified from samples and standard isolates DNA, by using HaeIII. M: molecular marker (100 bp); Lanes 1–13: samples; *Lm*: *Leishmania major *(IPAP/EG89/SI-177); *Lt*: *Leishmania tropica *(MHOM/TR/05/EP119); *Li*: *Leishmania infantum* EP50; NTC: non template control.

The ITS1-PCR was further identified by digestion with the restriction enzyme HaeIII. However, RFLP patterns for 32 positive samples analyzed presented restriction bands 60 and 200 bp (Figure 3) which are correlated to *L. tropica* (MHOM/TR/05/EP119) reference strain (positive control) pattern.

## 4. Discussion

The diagnosis of leishmaniasis in Syria is mostly based on clinical features and direct observation of the amastigotes stage in clinical materials [5, 6]. Many previous studies mentioned that the conventional methods are not able to differentiate between *Leishmania* species due to their homogeneous morphologies [14, 22, 23]. Therefore, it was necessary to apply a sensitive method helping in diagnosis, differentiate between different *Leishmania* species, and improve therapy procedure. 

This study focused on diagnosis and species identification of 32 samples obtained from patients with CL in Hama governorate in Syria. We used specific primers [21] that amplify a 300–350 bp fragment in the ribosomal internal transcribed spacer-1 (region that separates the ssu rRNA and 5.8S rRNA genes and varies between *Leishmania* species) from different samples onto glass slides. The ITS1 fragments which are similar to those obtained with *Leishmania* standard strains [15–17, 21, 24], were chosen as a target for diagnostic PCR analysis [16]. Many studies mentioned the important role of the restriction enzymes in the identification of all medically relevant *Leishmania* parasites [11, 16, 25]. As result of digestion with HaeIII, ITS1-PCR amplicon yielded 60 and 200 bp fragments which are corresponding with *L. tropica* patterns [15, 16, 24, 26]. It was confirmed that CL infections in all patients from Hama governorate were caused by *L. tropica*.

In conclusion, ITS1-PCR followed by RFLP can be considered the method of chance for diagnosing and identifying the causative species of cutaneous leishmaniasis, in Syria, for accuracy treatment.
